# Primary Epithelial Myoepithelial Carcinoma of Lung, Reporting of a Rare Entity, Its Molecular Histogenesis and Review of the Literature

**DOI:** 10.1155/2012/319434

**Published:** 2012-08-08

**Authors:** Farzana Arif, Susan Wu, Shahriyour Andaz, Stewart Fox

**Affiliations:** ^1^Department of Pathology, South Nassau Communities Hospital, One Healthy Way, Oceanside, NY 11572, USA; ^2^Department of Thoracic Surgery, South Nassau Communities Hospital, One Healthy Way, Oceanside, NY 11572, USA

## Abstract

Primary epithelial myoepithelial carcinoma of lung is a rare entity and is thought to arise from the submucosal bronchial glands distributed throughout the lower respiratory tract. Because of the rarity of this tumor, we describe one case of epithelial myoepithelial carcinoma arising in the bronchus intermedius and presenting as an endobronchial mass. A 57-year-old male patient presented with an incidental finding of an endobronchial mass located in the lumen of the right lower lobe bronchus and caused near total luminal occlusion of the bronchus. An endobronchial carcinoid tumor was entertained clinically. Subsequently the patient underwent an uneventful videothoracoscopic lobectomy of lower and middle lobes of the right lung. Morphologically and immunohistochemically the tumor was characterized by two cell populations with epithelial and myoepithelial cells forming duct-like structure. The final diagnosis of epithelial myoepithelial carcinoma of lung was rendered.

## 1. Case Presentation

**Figure 1:**
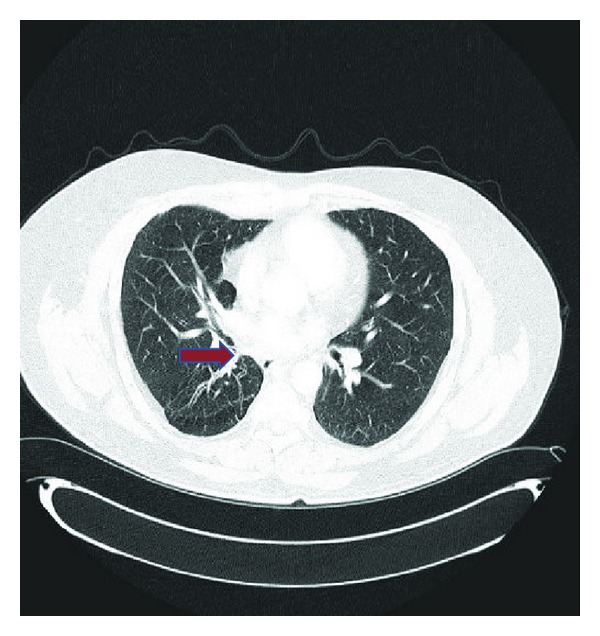
CT scan showing endobronchial mass (red arrow).

57-year-old male patient presented with colicky abdominal pain located at the previous incisional hernia site of the anterior abdominal wall. He had a history of smoking 1 cigarette pack per day for 20 years but quit over 10 years ago. His work up included a CT scan of the abdomen including slices from the lower chest. There was an incidental finding of an endobronchial mass of 2.0 centimeters in diameter and the mass almost entirely occluded the lumen of the right lower lobe bronchus. Patient denied any history of cough, shortness of breath, hemoptysis, fever, or weight loss. A PET/CT scan showed low-grade uptake with an SUV of 1.8 in the mass (Figure 1). Fibreoptic bronchoscopy showed a friable, smooth, and highly vascular looking mass in the right lower bronchus. Clinical diagnosis was endobronchial carcinoid tumor. The patient denied symptoms of a carcinoid syndrome, such as, flushing, diarrhea, wheezing, and cyanosis. Subsequently he underwent an uneventful videothoracoscopic right lower and middle lobectomy.

## 2. Materials and Methods

The tissue was fixed in 10% buffered formalin for 24 hours and embedded in paraffin. Sections were taken and stained with hematoxylin eosin. The immunohistochemical stains were performed at an outside facility, using the Leica polymer detection system Intense on the BondMax staining platform. The following antibodies and proteins were used: Cytokeratins AE1/AE3 (Invitrogen, Frederick MD; 1 : 400), CAM5.2 (BD Biosciences, San Jose, CA, USA; 1 : 200), CK7 (DAKO, Carpinteria, CA, USA; 1 : 8,000), CK903 (DAKO, Carpinteria, CA, USA; 1 : 250), S-100 (DAKO, Carpinteria, CA, USA; 1 : 10,000), Calponin (DAKO, Carpinteria, CA, USA; 1 : 2,000), SMA (BioCare Medical, Concord, CA, USA; 1 : 400), GFAP (Invitrogen, Frederick, MD, USA; 1 : 50), CD117 (DAKO, Carpinteria, CA, USA; 1 : 250), Synaptophysin (BioGenex, San Ramon, CA, USA; 1 : 400), Chromogranin (DAKO, Carpinteria, CA, USA; 1 : 4,000), Thyroglobulin (DAKO, Carpinteria, CA, USA; 1 : 50,000), TTF-1 (Thermo Scientific, Freemont, CA, USA; 1,000), CK20 (DAKO, Carpinteria, CA, USA; 1 : 2,000), BerEP4 (DAKO, Carpinteria, CA, USA; 1 : 400), B72.3 (Covance, Emeryville, CA, USA; 1 : 2,000), PSA (DAKO, Carpinteria, CA, USA; 1 : 16,000) and Ki-67 (DAKO, Carpinteria, CA, USA; ready-to-use (pre-diluted) and p27/Kip1 (DAKO, Carpinteria, CA, USA; 1 : 200) (Table 2).

## 3. Pathology

**Figure 2:**
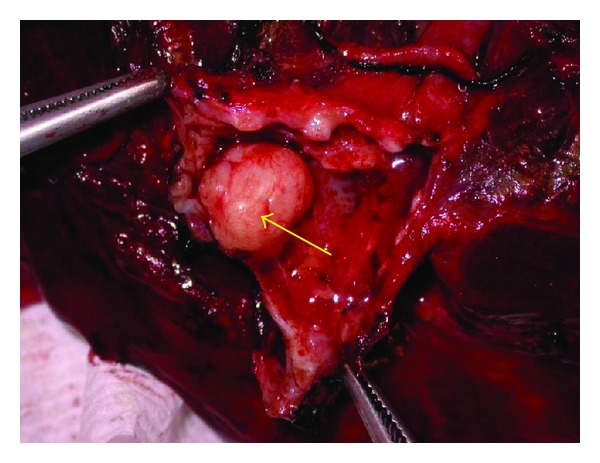
Gross, well-circumscribed endobronchial tumor.

**Figure 3:**
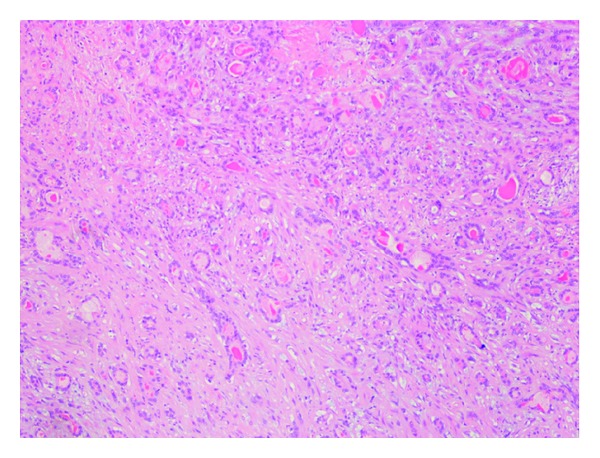
Tumor, duct-like structures and spindle areas (hematoxylin eosin, original magnification ×100).

**Figure 4:**
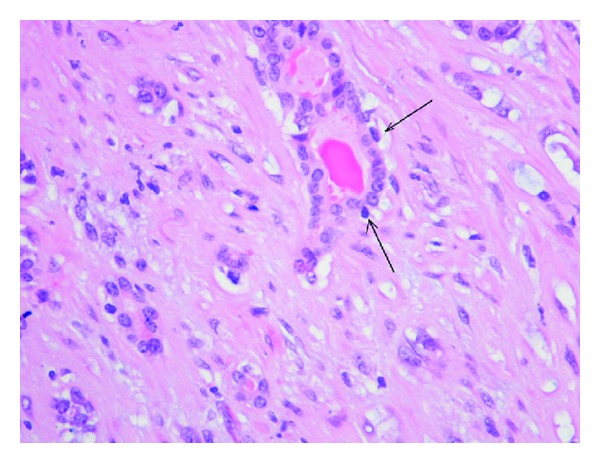
Tumor, inner glandular layer, and outer myoepithelial layer (hematoxylin eosin, original magnification ×400).

**Figure 5:**
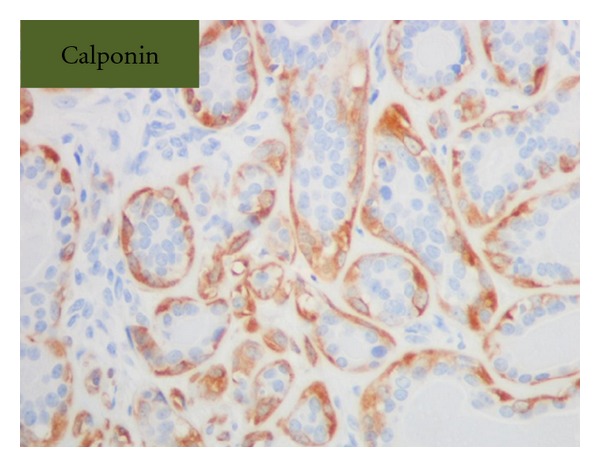
Myoepithelial stains, calponin, (original magnification ×400).

**Figure 6:**
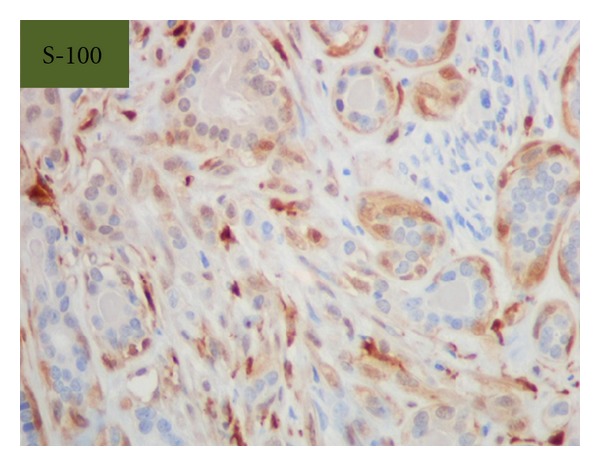
Myoepithelial stains, S-100 (original magnification ×400).

**Figure 7:**
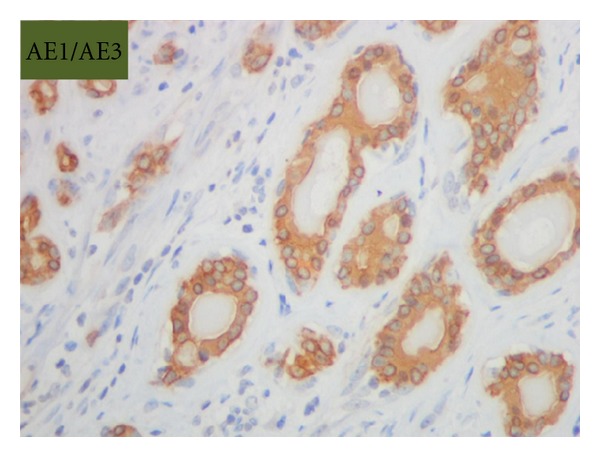
Epithelial stains, AE1/AE3 (original magnification ×400).

**Figure 8:**
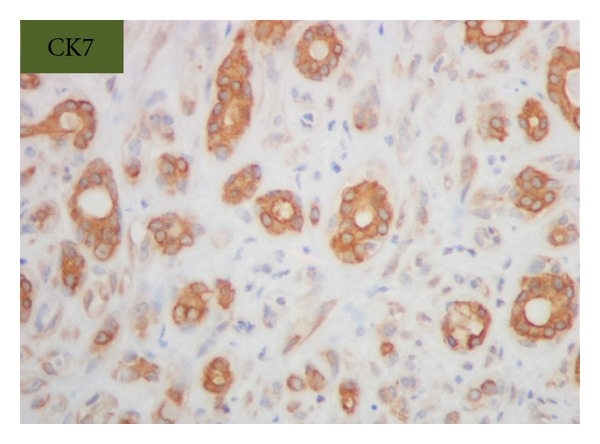
Epithelial stains, CK7 (original magnification ×400).

**Figure 9:**
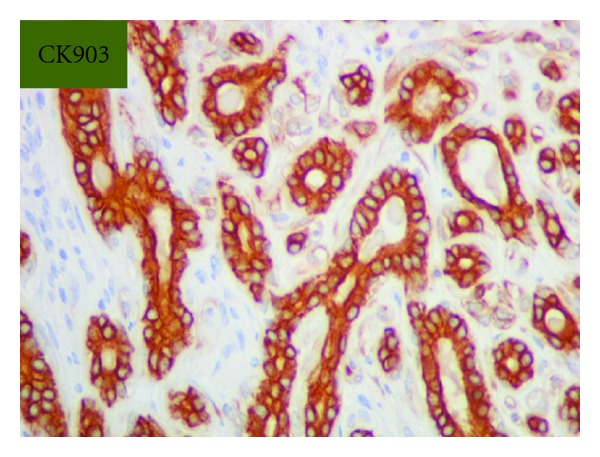
Epithelial stains, CK903 (original magnification ×400).

**Figure 10:**
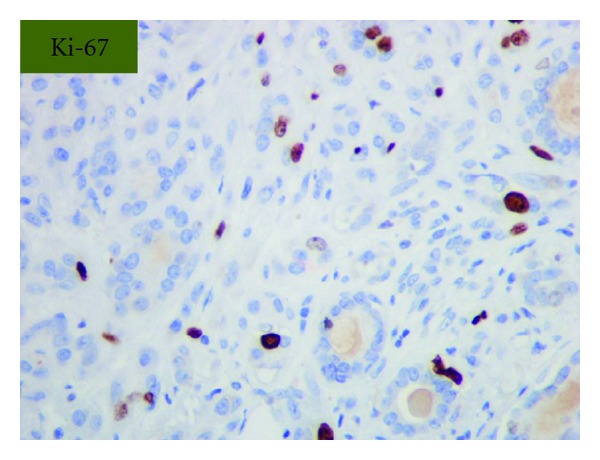
Ki-67 shows 2-3% proliferative activity (original magnification ×400).

Grossly, the tumor was a solitary, well-circumscribed, white-tan, polypoid, and unencapsulated mass located in the bronchus intermedius. It measured 1.2 × 1.0 × 0.8 cm in greatest dimension (Figure 2). Cut surface was solid whitish tan with no evidence of necrosis or hemorrhage. Microscopically, the mass was situated beneath the bronchial epithelium without capsule. The mass was composed of duct like structures, lined by two distinct cell layers. The inner cell layer was cuboidal cells with eosinophilic cytoplasm and centrally located nuclei and outer layer of cells with predominantly clear cytoplasm and uniform nuclei. The ducts like structures contained eosinophilic material in the luminal spaces. Some areas of the tumor showed more spindly cells with clear cytoplasm (Figures 3 and 4). The tumor showed 2-3 mitosis/10 high-power field (HPF). Immunohistochemical studies confirmed the biphasic nature of the tumor. The inner layer of ducts was reactive for cytokeratins (CAM5.2, AE1/AE3, CK7 and CK903) indicating epithelial cells differentiation, and outer layer of cells was reactive for S-100, calponin, SMA, GFAP suggestive of myoepithelial cells. CD117 showed positive reactivity in ducts and spindle cells (Figures 5, 6, 7, 8, and 9). The tumor did not show reactivity for synaptophysin, chromogranin, thyroglobulin, TTF-1 and CK20, BerEP4, B72.3, and PSA. Ki-67 showed 2-3% of proliferative activity (Figure 10). Immunostain for p27/kip-1 was performed and it showed nuclear reactivity in the epithelial component and minimal cytoplasmic positivity in the myoepithelial component (Table 2).

## 4. Discussion

Primary epithelial myoepithelial tumors of lung are rare neoplasms. These tumors are considered to be low-grade malignant neoplasms with histologic features similar to their counterparts of the salivary gland. Neoplasms with similar morphology are reported in the breast, skin, and lacrimal glands [12–14]. It is a relatively less common entity that had only been recognized within the last 2 decades [12]. The WHO defines this entity as “a malignant tumor composed of variable proportions of two-cell types, which typically form duct like structures. The biphasic morphology is represented by an inner layer of duct lining, epithelial type cells, and an outer layer of clear myoepithelial cells” [15]. To date, 24 cases have been reported in the lung, variably classified as “adenomyoepithelioma”, “pneumocytic adenomyoepithelioma”, “myoepithelioma”, “epithelial myoepithelial carcinoma”, and epithelial myoepithelial tumor. One suggested term by Pelosi was pulmonary epithelial myoepithelial tumor of unproven malignant potential (PEMTUMP) [7, 12].

A review of the literature reveals a total of 25 cases including the current case in the last 18 years. Out of 25 cases, there is slightly female predominance, 15 are females and 10 are males. Age range is 34–76 years and the average age is 54 years. 13 cases arise on the right side, 11 arise on left side, and one case does not state the site. The size of the tumor ranges from 0.8 cm to 16 cm; with average of 3.2 cm. Sizes are not available in two cases. Most of the cases are presented as polypoid endobronchial mass. Grossly these tumors are not encapsulated but well-delineated masses, and they present as an exophytic intrabronchial growth pattern, sometimes completely obstructing the bronchial lumina [13]. One recent case is described as being intraparenchymatous mass without apparent bronchial or visceral pleura connection [13]. All cases show characteristic morphology with biphasic pattern of an inner layer of epithelial cells immunoreactive for cytokeratins and an outer layer of myoepithelial cells immunoreactive for S-100 and smooth muscle actin (SMA). Few cases show a solid component with spindle and clear cells including our case, and the solid component in most of the cases shows positive reactivity for myoepithelial markers. A PAS-positive, diastase-sensitive amorphous material is observed in the intercellular spaces and luminal spaces of duct-like structures. Necrosis is rare, but it has been described [15]. Ki-67 labeling is reported in few cases and it ranges from <1% to 12%. Since there are only few cases reported in the literature with limited followup, the overall approach is to complete excision of these tumors because of infiltrating morphologic pattern. So far there is only one case that showed extensive lymphovascular, perineural invasion, and lymph node involvement and local metastasis [12]. Followup of 22 out of 25 cases ranges from 4 months to 7 years. No followup is available in three cases including one case lost to followup. The rest of cases reported no recurrence or distant metastasis (Table 1). The disease-free survival and lack of nodal involvement in majority of cases correlate well with the lack of recognized histopathologic features of aggressiveness [8].

The biologic behavior of this type of tumor is still unclear. Attempts were made by Pelosi et al. to find out the changes at molecular level and further supported by Munoz et al. [13] Protein p27/kip-1 is a cyclin-dependent kinase inhibitor (CDK) that blocks cell cycle in G0 and G1. It is present in high concentration in quiescent cells and its levels slowly decrease while cells are stimulated to begin the cell cycle. Thus, p27/kip-1 inhibits and controls the progression of the cell cycle and therefore exercises a function of inhibition of tumorigenesis; in fact, it has been demonstrated that levels of p27/kip-1 are decreased in many tumors. Moreover, Besson et al. have recently described a dual function of this protein; as well as acting as an inhibitor of tumorigenesis, it would have oncogenic functions when it presents cytoplasmic location, acting through mediators which are little known [16]. The work also suggests that p27/kip-1 oncogenic activity leads to aberrant stem and progenitor cell expansion in the lung and retina. This study provides the first direct in vivo evidence that in addition to its role as a tumor suppressor, p27/kip-1 also functions as an oncogene. So, in epithelial-myoepithelial tumors of lung, in accordance with Pelosi et al. [7], an aberrant subcellular location of p27/kip-1 into the myoepithelial cell would provoke the loss of its growth-inhibition function through the lack of restriction of proliferation of myoepithelial component. Munoz et al. support this concept along with proved oncogenic function of this protein [13]. In our case, nuclear epithelial positivity is more pronounced. There is minimal cytoplasmic positivity seen in the myoepithelial component which verifies this concept, but evaluation of more cases is needed to support this concept. In conclusion, epithelial-myoepithelial tumor is a neoplasia of uncertain malignant potential, which can arise exceptionally in the lung.

Epithelial myoepithelial carcinoma is not difficult to diagnose in resection specimen because of characteristic morphology and immunohistochemical studies. Only in very scant biopsy material or sampling of areas without the characteristic bilayered tubules may cause difficulty and confusion with other neoplasms [12].

The differential diagnosis can be extensive and in many occasions it is going to depend on the relative predominance of myoepithelial component or on the biphasic pattern. If the tumor has predominantly biphasic pattern, the differential diagnosis includes pulmonary pleomorphic adenoma. If there is predominance of clear cells (myoepithelial cells) then myoepithelioma, myoepithelial carcinoma, and clear cell “sugar” tumor are in differential diagnosis. Clear cell “sugar” tumor can be differentiated on immunohistochemical stains since these tumors show positivity for melanocytic markers like HMB-45 and negativity for cytokeratins. If a tumor shows infiltrating pattern with some solid areas, mucoepidermoid carcinoma, acinic cell carcinoma, adenoid cystic carcinoma, and metastatic renal cell carcinoma are other differentials to consider. Surgical resection appears to be the treatment of choice by any of the current surgical modalities and depending on the clinical setting.

## Figures and Tables

**Table 1:** 

Year	Case author	Age	Sex	Diagnosis	Site	Siz (cm)	Lymph node	Met	Outcome
1994	Nistal et al. [1]	55	F	EMT	RUL	2	N/A	Neg	FOD at 2 yr
1993	Hornouchi [2]	57	F	EMT	Trachea	2.2			N/A
1995	Tsuji et al. [3]	66	M	Adenomyo epithelioma	R intermedius bron	1.6			AW after 36 months now died of unrelated disease
1997	Wilson and Moran [4]	55	F	EMC	LLL	3.9			AW after 7 m
1998	Shanks et al. [5]	67	M	EMC	LLL	1.3			Lost to follow up
1998	Ryska et al. [6]	47	F	EMC	RULB	N/A			AW after 13 m
2001	Pelosi et al. [7]	47	M	EMT of unprovn malig potential	LULB	1.5			AW after 6 months
2001	Fulford et al. [8]	1 : 55	F	EMC	RMB	5			FOD at 8 months
		3 : 56	M	EMC		N/A			FOD at 5 yrs
		4 : 57	F	EMC	LMB	1.5			FOD at 8 yrs
		5 : 54	F	EMC	RUL	1.5			FOD at 7 yrs
2003	Doganay et al. [9]	73	M	EMC	LLLB	5			AW after 34 m
2004	Ru et al. [10]	73	M	EMC	LULB	3.8			FOD at 8 months
		1 : 54	F	Pneumocytic	RLL	2.6			FOD at 31 months
		2; 62	F	adenomyoepithelioma	LLL	2			FOD at 14 months
2007	Chang et al. [11]	3; 58	F		RML	1.2			FOD at 13 months
		4; 57	F		LUL	0.8			FOD at 78 months
		5; 52	F		RUL	1.2			FOD at 5 months
		1; 38	M	EMC	LLL	5	One case		FOD at 4 months
		2; 48	M	EMC	RUL	2.5	shows lymph		FOD at 12 months
2009	Nguyen et al. [12]	3; 52	F	EMC	LLL	3	node inv		N/A
		4; 54	M	EMC	RUL	3			FOD at 12 months
		5; 56	F	EMC	L main bronchus	4.2			FOD at 2 months
2011	Munoz et al. [13]	76	F	EMC	RUL	2.7			N/A
current case		57	M	EMC	Right bronchus intermedius	1.2			FOD at 9 months

EMC: epithelial myoepithelial carcinoma, EMT: epithelial myoepithelial tumor, RUL: right upper lobe, LLLB: left lower lobe bronchus, RULB: right upper lobe bronchus, LULB: left upper lobe bronchus, RMB: right main bronchus, RLL: right lower lobe, RML: right middle lobe, RUL: right upper lobe, LLL: left lower lobe, LUL: left upper lobe, LMB: lower main bronchus, FOD: free of disease, AW: alive with, N/A: not available.

**Table 2:** 

	CAM5.2	AE1/AE3	CK7	CK903	S-100	Calponin	SMA	CD117	GFAP
Source and dilution	BD Biosciences, San Jose, CA, USA; 1 : 200	Invitrogen, Frederick, MD, USA; 1 : 400	DAKO, Carpinteria, CA, USA; 1 : 8000	DAKO, Carpinteria, CA, USA; 1 : 2000	DAKO, Carpinteria, CA, USA; 1 : 10000	DAKO, Carpinteria, CA, USA; 1 : 2000	BioCare Medical, Concord, CA, USA; 1400	DAKO, Carpinteria, CA, USA; 1 : 250	Invitrogen, Frederick, MD, USA; 1 : 50
Epithelial cell	Positive	Positive	Positive	Positive	Negative	Negative	Negative	Negative	Negative
Myoepithelial cells	Negative	Negative	Negative	Negative	Positive	Positive	Positive	Positive	Positive
